# Mitochondrial dysfunction: A notable contributor to the progression of Alzheimer's and Parkinson's disease

**DOI:** 10.1016/j.heliyon.2023.e14387

**Published:** 2023-03-11

**Authors:** Abolaji Samson Olagunju, Foysal Ahammad, Abiola Adeyanju Alagbe, Titilayomi Ayomide Otenaike, John Oluwafemi Teibo, Farhan Mohammad, Ahad Amer Alsaiari, Olabode Omotoso, Md Enamul Kabir Talukder

**Affiliations:** a Department of Immunology, Institute of Biomedical Sciences, University of Sao Paulo, Brazil; b Division of Biological and Biomedical Sciences, College of Health and Life Sciences, Hamad Bin Khalifa University, Doha, Qatar; c Department of Human Anatomy, University of Port Harcourt, Nigeria; d Department of Genetics and Molecular Biology, Universidade Federal Do Rio Grande Do Sul, Porto Alegre, Brazil; e Department of Biochemistry and Immunology, Ribeirão Preto Medical School, University of São Paulo, Ribeirão Preto, SP-Brazil, Av Bandeirantes, 3900, 14049- 900, Ribeirão Preto, SP, Brazil; f Department of Clinical Laboratory Sciences, College of Applied Medical Sciences, Taif University, Taif, Saudi Arabia; g Department of Biochemistry, College of Medicine, University of Ibadan, Nigeria; h Department of Genetic Engineering and Biotechnology, Jashore University of Science and Technology, Jashore, Bangladesh

**Keywords:** Mitochondrial dysfunction, Alzheimer's disease, Parkinson's disease, Neurodegeneration, Amyloid β, Parkin

## Abstract

Mitochondrial dysfunction remains a pivotal mechanism in manifold neurodegenerative diseases. Mitochondrial homeostasis within the cell is an essential aspect of cell biology. Mitochondria, the power-generating organelle of the cell, have a dominant role in several processes associated with genomic integrity and cellular equilibrium. They are involved in maintaining optimal cell functioning and ensuring guidance against possible DNA damage, which could lead to mutations and the onset of diseases. Conversely, system perturbations, which could be due to environmental factors or senescence, induce changes in the physiological balance and result in mitochondrial function impairment.

As a result, we present a general overview of the pathological pathways involved in Alzheimer's and Parkinson's diseases caused by changes in mitochondrial homeostasis. The focal point of this review is on mitochondrial dysfunction being a significant condition in the onset of neuronal disintegration. We explain the pathways associated with the dysfunction of the mitochondria, which are common among the most recurring neurodegenerative diseases, including Alzheimer's and Parkinson's disease. Are mitochondrial dysfunctions an early event in the progression of neuropathological processes? We discovered that mtDNA mutation is a major contributor to the metabolic pathology of most neurological disorders, causing changes in genes important for physiological homeostasis. As a result, genetic changes in presenilin, Amyloid-, ABAD, DJ-1, PINK-1, PARKIN, alpha-synuclein, and other important controlling genes occur. Therefore, we suggest possible therapeutic solutions.

## Graphical abstract

**Figure:**
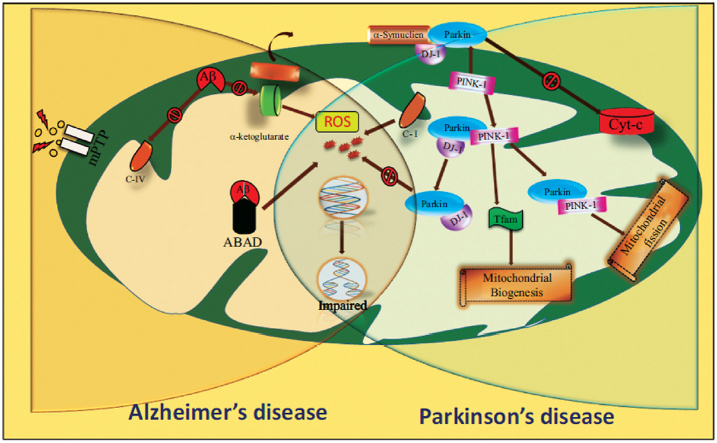

## 1 Introduction

Mitochondria is a significant constituent of the cell, sitting at the edge of cellular metabolism, energy production, and cell death. These distinct roles of mitochondria need a host of proteins working both in an inner and outer mitochondrial membrane. Proteomics has mapped over 1000 constituents to the mitochondria [1,2] even though the mitochondrial genome only encodes for 13 genes. This shows that a major percentage of cellular factors partake in mitochondrial biology.

Classically, mitochondria are known as the powerhouse of the cell, and the active core producing ATP (adenosine triphosphate) for cellular activities. Thorough research on the functions and morphology of mitochondria revealed that they perform numerous roles. Remarkably, mitochondria have their genome, with the mitochondrial DNA (mtDNA) enclosed in the nucleoids in close relationship with the inner membrane of the mitochondria [44]. Mutations in the mtDNA are responsible for several human diseases generally known as mitochondrial disorders [3]. Also, mitochondria are dynamic and essential organelles with the capability to change their size and shape to respond to various cellular demands and preserve cellular homeostasis. For the cell homeostasis maintenance, mitochondria play a vital role in determining cell fate due to their involvement in regulating programmed cell death. Mitochondria are very sensitive to all subtle alterations disturbing the cell homeostasis which then alter their number and shape. The mechanisms of fusion and fission are essential, to increase their number or to repair a damaged mitochondrion, as in case of enlarged demand of energy or to enhance their exclusion when damaged to maintain cellular integrity.

**Fig. 1:**
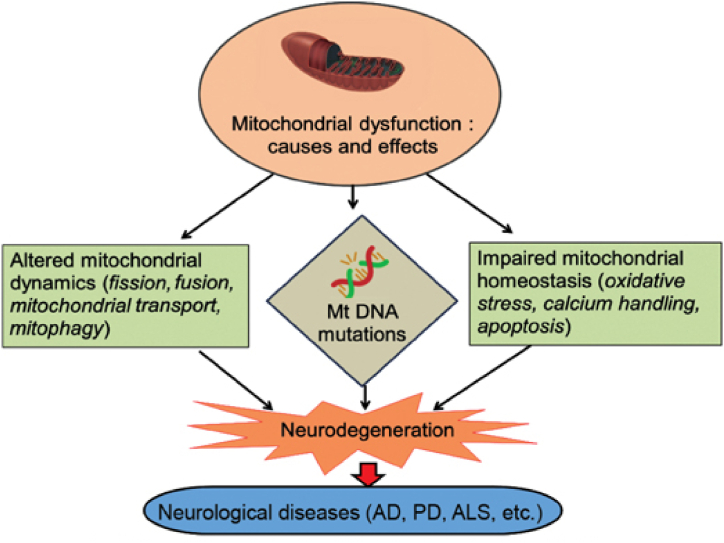
Causes and role of mitochondrial dysfunction in neurodegenerative disease [44].

The dysfunction of mitochondria is related to some developmental and age-related diseases, mostly neurodegenerative diseases for example Alzheimer's, Parkinson's, and Huntington's disease, and amyotrophic lateral sclerosis [Fig. 1] [4].

**Fig. 2:**
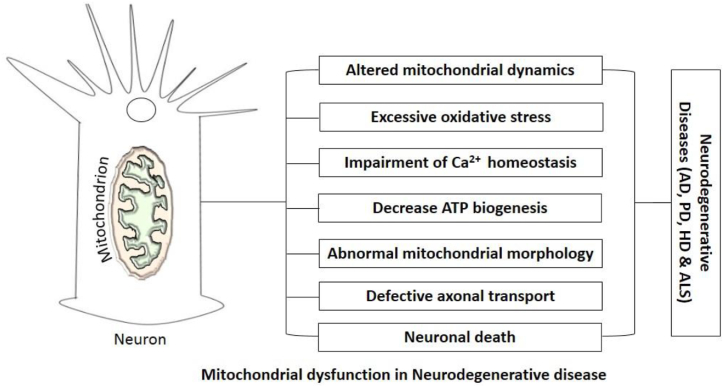
Mitochondrial dysfunction in neurodegenerative disease [8].

Neurodegeneration results in the loss of function and structure of the neuronal system. The hallmarks of most neurodegenerative diseases involve abnormal accumulation and folding of proteins inside the neuronal cell bodies. These alterations in the protein metabolism commonly result in dysfunction and neuronal cell death in the central nervous system (CNS) [5]. The consequence of mitochondrial dysfunction in the development of neurodegeneration is captivating. MtDNA carries out numerous metabolic processes including the Krebs cycle and the respiratory chain to generate energy. Most of the time, the circular mtDNA accumulates mutations as the individual ages and these could result in mitochondrial dysfunction, capable of initiating many complications in the human body, such as the development and progression of neurodegenerative diseases [6] and[46] . The main reason for mitochondrial dysfunction is undecided; although, mtDNA mutation, oxidative damage and, accumulation of mitochondrial proteins resulting in abnormal mitochondrial morphology have been implicated. Since oxidative stress can destroy mitochondrial protein, lipids, and nucleic acid and this organelle is involved in the generation of intracellular reactive oxygen species (ROS), which can result in mtDNA mutations [7, 41]; this makes oxidative stress be a critical factor, responsible for mitochondrial dysfunction (Fig. 2 [8]).

### 1.1 General pathomechanisms of Alzheimer's and Parkinson's disease

**Fig. 3:**
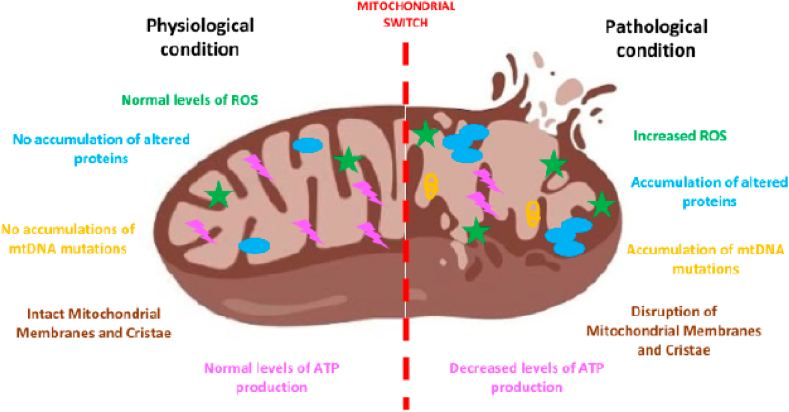
Mitochondrial switch in neurodegeneration [10].

Neurodegenerative diseases comprise of heterogeneous disorders, described by the continuous loss of definite neuronal populaces and circuits in the CNS activated by mitochondrial dysfunctions [7,9]. Generally, there is a switch in mitochondrial function [Fig. 3] [10], in neurodegenerative diseases contributing significantly to the change to a degenerative condition from a normal physiological one. The aggregation of diverse stresses and the parallel aberration of series of cell-protective processes stimulate neurodegeneration.

The Amyloid hypothesis states that amyloid-β deposition and Tau protein which is an important protein for microtubule assembly is accountable for Alzheimer's disease pathogenesis. Beta (β) and gamma (γ)-secretase act on the Amyloid precursor protein (APP) and then cleave APP in amyloid-β (Aβ) peptides in sizes 38, 40, 42 of amino acids length. Outside the neurons, the largest fragment that is Amyloid-β-42 accumulates and oligomerizes which results in the formation of amyloid plaques [11]). However, through competitive binding, Amyloid-β-40 interacts with Amyloid β-42 in the monomeric and non-toxic states. It was then observed that Amyloid β-40 inhibits Amyloid β-42 fibril formation and also reduced the levels of Amyloid β-40 associated with the formation of plaque in Amyloid precursor protein transgenic mice. Meanwhile, Amyloid β-40 has a protective role in Alzheimer's disease [12]. Tau protein becomes phosphorylated in the neurons and then interacts with the other tau protein threads resulting in the development of neurofibrillary tangles mostly in the pyramidal cells.

Hyper-phosphorylated tau proteins interact with the microtubule, which is responsible for the function and shape of neurons. Hyper-phosphorylation of Tau and aggregation are figured by Amyloid-β accumulation. Therefore, Amyloid-β aggregation was established to start a neurodegenerative cascade resulting in loss of a neuron and dementia known as the amyloid cascade hypothesis. Alzheimer's disease results from mutations in the Amyloid precursor protein, Presenilin (PS) −1 or −2 on chromosomes 21, 14 and 1 respectively [38] which increases Amyloid-β deposition.

The markers of Parkinson's disease comprise of the presence of intra-neuronal lewy bodies and dopaminergic neurodegeneration with advanced Parkinson's disease cases showing a neuronal loss in the brainstem, sub-cortex, cortex and, peripheral autonomic sites. Mostly, Parkinson's disease is linked to genetic modifications such as E46K, A30P and, A53T mutations in gamma (γ) -synuclein, triplication of non-mutant gamma (γ)-synuclein resulting in excessive pathological protein buildup. Furthermore, Leucine-rich repeat kinase-2 (LRRK2), protein deglycase (DJ-1), PTEN-induced kinase-1 (PINK1) mutations culminate in mitochondrial dysfunction and also increase oxidative stress which then reduces the level cellular ATP involved in reducing proteasomal activity with low clearance of accumulated proteins. Likewise, parkin and ATP13A2, (gene that encodes lysosomal ATPase) mutations also disrupt the cellular protein breakdown machinery [13].

The double hit hypothesis affirms that Parkinson's disease progresses as an interaction between environmental factors and mutated genetic. Moreover, excitotoxicity is a fundamental feature for the development of Parkinson's disease. Hence, a combination of the damaging properties of multiple genetic mutations eventually results in pathological protein aggregation, reduced synthesis of ATP and, also dopaminergic neuronal death [11].

### 1.2 Alzheimer's disease (AD) and mitochondrial dysfunction

By 2040, Alzheimer's disease (AD) which is the commonest and devastating neurodegenerative disease has been predicted to affect over eighty-one million people globally. AD's pathology is distinguished by senile plaques, mostly comprising of accumulated β-amyloid and intracellular neurofibrillary tangles generated by the aggregation tau protein. In the progression of AD, two major players are mostly involved and these are; tangles and plaques [14].

Increased buildup of extra-neuronal amyloid plaques, resulting from the proteolytic processing of the APP (amyloid precursor protein) and NFTs (intra-neuronal neurofibrillary tangles), generated by hyper-phosphorylated tau protein (pTau) are typically examined in the brain cells of Alzheimer's individuals [15]. Plaques consist of amyloid-β fibrils that gather from oligomeric and monomeric intermediates and are prognostic markers of AD. Interestingly, the early stage of Alzheimer's is detected by functionally and morphologically impaired mitochondria susceptible to increase production of reactive oxygen species (ROS), leading to a decrease in brain energy due to the reduction of ATP [16].

Once Amyloid-β is formed, it is capable of communicating with the mitochondria leading to additional mitochondrial aberrations. Amyloid-β overwhelms complex IV and α-ketoglutarate dehydrogenase and then binds to the mitochondrial matrix protein (Amyloid-β- binding alcohol dehydrogenase- ABAD). When the communication between Amyloid-β and alcohol dehydrogenase-ABAD) is inhibited, it indicates diminished Amyloid-β induced neuronal apoptosis and free radical generation. Amyloid-β prevents two major mitochondrial enzymes (α-ketoglutarate dehydrogenase and cytochrome oxidase) which are observed to be at a low level in the brains of subjects with AD. At the mitochondrial level, cells are more delicate to the apoptotic stimuli formed because of presenilin mutations. Presenilin forms an active gamma (γ)- secretase complex through interaction with Nicastrin (NCT), Anterior pharynx-defective (APH)-1 and, Presenilin enhancer (PEN)-2 and also responsible for breaking β-APP.

**Fig. 4:**
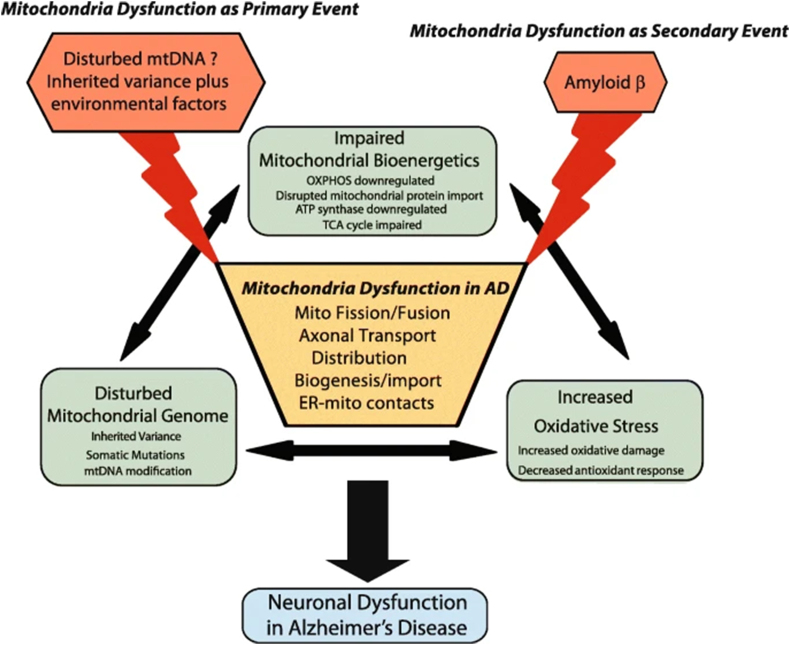
Critical contribution of mitochondrial dysfunction in AD [[19], [45]].

The first proof of the pathogenic mechanisms in AD is the progressive extracellular aggregation of β-amyloid peptide (Aβ) in the brain and neurofibrillary tangles of the hyper-phosphorylated tau proteins inside neurons, influencing the advanced loss of hippocampal and cortical neurons and then initiating brain atrophy, followed by cognitive and memory loss [17]. Decrease activity in complex IV has been established in mitochondria from the hippocampus and platelets of Alzheimer's patients and in AD cybrid cells [18]. A buildup of Amyloid-β results in mitochondrial dysfunction, energy failure and, oxidative stress (Fig. 4 [[19], [45]]), before the progression of plaque pathology. Before amyloid and tau deposition, oxidative stress-induced damage takes place, and mitochondrial dysfunctions are initial events preceding neurodegeneration.

Mitochondria look functionally and morphologically modified, impacting on many processes for example excess formation of ROS, leading to a reduction in brain energy due to decreased level of ATP, change in calcium homeostasis, and also apoptosis induction [20,21].

### 1.3 Parkinson's disease and mitochondrial dysfunction

Parkinson's disease (PD), the second most common neurodegenerative disorder described by loss of dopaminergic neurons progressively in the substantia nigra pars compacta (SNpc), resulting in diminished motor function linked to α-synuclein aggregates (Lewy bodies) [14]). PD patients demonstrate a progressive rigidity and tremors of the muscle due to a low level of dopaminergic modulation on striatal neurons modifying motor systems [42]. Other markers of this disease include the presence of immuno-reactive α-synuclein and ubiquitin and inclusions of lewy bodies. With over 680,000 people in North America as of 2010 [22], PD represents an increasing condition with an impact on approximately 10 million people globally.

For more than 20 years, studies of human patient mutations and cell biology have shown a surveillance model for mitochondrial through Parkin (E3 ubiquitin ligase) and PINK1 (PTEN-induced kinase 1) vital to support healthy neurons [11]). In healthy mitochondria, PINK1 is translated into the outer mitochondrial membrane (OMM) and then translocated into the mitochondria for proteolytic degradation. This shows that in healthy mitochondria, PINK1 levels are usually low. During mitochondrial impairment for instance membrane depolarization, PINK1 is not degraded but then exists as a membrane-anchored component in the outer mitochondrial membrane (OMM). In its new localization, Parkin is activated through PINK1-mediated phosphorylation. Upon activation, mitophagy is initiated with Parkin-mediated ubiquitination signals, which is the selective degradation of mitochondria through the autophagosome.

Mitochondrial dysfunctions have been associated with both familial and sporadic PD and are linked to conflicts of mitochondrial dynamics, morphology, and function [23]. High levels of reactive oxygen species, owing to the high metabolic call, control the build-up of toxic oxidative species and structural modifications of complex I, altering mitochondria functionality in brains of both human and mouse models [24]. Furthermore, the accumulation of alpha (α)-synuclein oligomers initiates mitochondrial membrane permeabilization and direct toxicity via increase in the formation of reactive oxygen species and therefore resulting in neuronal death. Instabilities in calcium homeostasis and overload might initiate the mitochondrial permeabilization transition pore (MPTP) opening, leading to reactive oxygen species production, cytochrome C release, and induction of apoptosis.

In PD, mitochondrial dysfunctions also result in changes in mitochondria biogenesis due to transcription factors dysregulation for example the peroxisome proliferator-activated receptor (PPAR)- gamma coactivator 1-alpha (PGC1α) . Additionally, mitochondrial fragmentation takes place swiftly after the loss of membrane potential was also observed in Parkinson's disease subjects. Also, fission and fusion proteins for example the dynamin-related protein 1 (DRP1) are transformed in familial PD model . Remarkably, genes like PINK-1 and Parkin, that are connected with Familial-Parkinson's disease partake in the regulation of mitochondrial dynamics and many other genetic mutations, such as Parkin, PINK-1, LRRK2, DJ-1, and α-Synuclein, have been related to familial PD and the resultant gene products also partake in mitophagy [25].

**Fig. 5:**
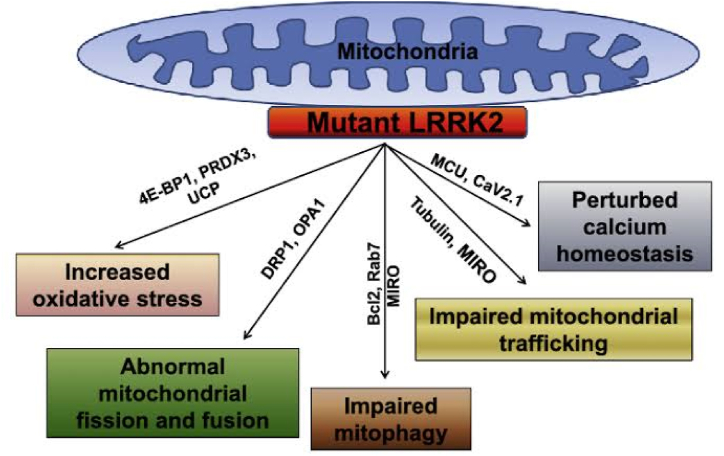
Molecules and pathways affecting the functions of Mitochondria [26].

Both Parkinson's and Alzheimer's disease involves modification in the mtDNA. Alpha-synuclein is the main protein in Parkinson's pathogenesis. Over-expression of the alpha (α)-synuclein leads to mitochondrial dysfunction and oxidative stress. Parkin is connected to the OMM, which then inhibits mitochondrial swelling, cytochrome *c* release and caspase activation. In the proliferating cells, Parkin moves inside and with the mitochondrial transcription factor A (TFAM) leading to mitochondrial biogenesis. Parkin acts downstream to the PINK-1 and mutations in these results in excessive mitochondrial fission and reduced fusion. DJ-1 acts as a redox sensor and protects against oxidative stress by acidifying its cysteine residue. LRRK2 mutation also leads to excessive mitochondrial fission. Complex activity is reduced resulting in oxidative stress (Fig. 5 [26]) [8].

### 1.4 Recent approaches and potential therapies

The clinical complexity and genetic change caused by mutation(s), heteroplasmy (the presence of both mutant and normal genes), environment, and age are the primary causes of mitochondrial dysfunction or disease [27]. The complexity of neurodegenerative diseases is best addressed through prevention and treatment. Currently, there is no ultimate treatment due to the multifactorial effects of mitochondrial dysfunction beyond the neurons and brain. Therapeutic drugs, for instance, resveratrol [43], luteolin [19, 32], and quercetin [28], to induce mitochondrial biogenesis and initiate a diversity in the metabolic target of mitochondrial transcription factors [29], are being considered in several studies. These natural antioxidant phytochemicals are present in plants used as food sources such as carrots, berries, red wine, and oregano [30].

Recently, the use of microRNAs (miRNAs) in the regulation of mitochondrial processes is gaining awareness, as miRNA-based strategies are used as potential therapeutics [31].

## 2 Conclusion

Mitochondrial dysfunction is a common attribute of neurodegeneration. Any dysfunction in it results in the pathogenesis of several disorders. Certainly, mitochondrial dysfunctions emerge early in neurodegeneration and, their aggregation induces or exacerbates the degenerative cascade affecting neuronal tissue. Mitochondrial dysfunction and oxidative stress occur early in Alzheimer's and Parkinson's disease. There is testimony that it has a chief role in the pathogenesis of these diseases. Many of these proteins interact with the mitochondria such as Amyloid-β, presenilin, ABAD, DJ-1, PINK-1, PARKIN, and alpha-synuclein are targets for many pharmacological interventions.

## Author contribution statement

All authors listed have significantly contributed to the development and the writing of this article.

## Funding statement

This work was partly supported by intramural funding to MF from the College of Health and Life Science (CHLS), Hamad Bin 10.13039/501100004070Khalifa University, 10.13039/100007458Qatar Foundation. FA received support from the College of Health and Life Sciences (CHLS), Hamad Bin 10.13039/501100004070Khalifa University.

## Data availability statement

No data was used for the research described in the article.

## Declaration of competing interest

The authors declare no competing interests.

## List of abbreviations

DNA: Deoxyribonucleic acid
mtDNA: mitochondrial DNA
CNS: Central Nervous System
ROS: Reactive Oxygen Species
APP: Amyloid precursor protein
PS: Presenilin
LRRK2: Leucine-rich repeat kinase-2
DJ-1: protein deglycase
PINK1: PTEN-induced kinase-1
ATP: Adenosine triphosphate
AD: Alzheimer's Disease
PD: Parkinson's Disease
NFT: Neuronal neurofibrillary tangles
pTau: Phosphorylated tau protein
ABAD: Amyloid-β- binding alcohol dehydrogenase
NCT: Nicastrin
APH: Anterior pharynx-defective
PEN: Presenilin enhancer
SNpc: substantia nigra pars compacta
OMM: outer mitochondrial membrane
MPTP: mitochondrial permeabilization transition pore
PPAR: peroxisome proliferator-activated receptor
PGC1α: peroxisome proliferator-activated receptor (PPAR)- gamma coactivator 1-alpha
DRP1: dynamin-related protein 1
TFAM: Mitochondrial transcription factor A

## References

[1] Hung V., Lam S.S., Udeshi N.D., Svinkina T., Guzman G., Mootha V.K., Carr S.A., Ting A.Y. (2017). Proteomic mapping of cytosol-facing outer mitochondrial and ER membranes in living human cells by proximity biotinylation. Elife.

[2] Calvo S.E., Clauser K.R., Mootha V.K. (2016). MitoCarta2.0: an updated inventory of mammalian mitochondrial proteins. Nucleic Acids Res..

[3] Steele H.E., Horvath R., Lyon J.J., Chinnery P.F. (2017). Monitoring clinical progression with mitochondrial disease biomarkers. Brain.

[4] Rashid Waseem, Anas Shamsi, Kazim N., Syed, Islam Asimul (2021). An insight into mitochondrial dysfunction and its implications in neurological diseases. Curr. Drug Targets.

[5] Reddy P.H., Manczak M., Kandimalla R. (2017). Mitochondria-targeted small molecule SS31: a potential candidate for the treatment of Alzheimer's disease. Hum. Mol. Genet..

[6] Kandimalla R., Manczak M., Yin X., Wang R., Reddy P.H. (2018). Hippocampal phosphorylated tau induced cognitive decline, dendritic spine loss and mitochondrial abnormalities in a mouse model of Alzheimer's disease. Hum. Mol. Genet..

[7] Yin X., Manczak M., Reddy P.H. (2016). Mitochondria-targeted molecules MitoQ and SS31 reduce mutant huntingtin-induced mitochondrial toxicity and synaptic damage in Huntington's disease. Hum. Mol. Genet..

[8] Kumar A., Dhawan A., Kadam A., Shinde A. (2018). Autophagy and mitochondria: targets in neurodegenerative disorders. CNS Neurol. Disord. - Drug Targets.

[9] Kandimalla R., Manczak M., Yin X., Wang R., Reddy P.H. (2018). Hippocampal phosphorylated tau induced cognitive decline, dendritic spine loss and mitochondrial abnormalities in a mouse model of Alzheimer's disease. Hum. Mol. Genet..

[10] Wang Y., Liu N., Lu B. (2019). Mechanisms and roles of mitophagy in neurodegenerative diseases. CNS Neurosci. Ther..

[11] Wu Y., Chen M., Jiang J. (2019). Mitochondrial dysfunction in neurodegenerative diseases and drug targets via apoptotic signaling. Mitochondrion.

[12] Yan Y., Wang C. (2007). Aβ40 protects non-toxic Aβ42 monomer from aggregation. J. Mol. Biol..

[13] Pan T., Kondo S., Le W., Jankovic J. (2008). The role of autophagylysosome pathway in neurodegeneration associated with Parkinson's disease. Brain.

[14] Tan S.H., Karri V., Tay N.W.R., Chang K.H., Ah H.Y., Ng P.Q., Ho H.S., Keh H.W., Candasamy M. (2019 Mar). Emerging pathways to neurodegeneration: dissecting the critical molecular mechanisms in Alzheimer's disease, Parkinson's disease. Biomed. Pharmacother..

[15] Reddy P.H., Beal M.F. (2008). Amyloid beta, mitochondrial dysfunction and synaptic damage: implications for cognitive decline in aging and Alzheimer's disease. Trends Mol. Med..

[16] Gomes C.M., Santos R. (2013). Neurodegeneration in Friedreich's ataxia: from defective frataxin to oxidative stress. Oxid. Med. Cell. Longev..

[17] Herholz K. (2012). Use of FDG PET as an imaging biomarker in clinical trials of Alzheimer's disease. Biomarkers Med..

[18] Du H., Guo L., Yan S., Sosunov A.A., McKhann G.M., Yan S.S. (2010). Early deficits in synaptic mitochondria in an Alzheimer's disease mouse model. Proc. Natl. Acad. Sci. U.S.A..

[19] Wang B., Lu Y., Wang R., Liu S., Hu X., Wang H. (2020). Transport and metabolic profiling studies of amentoflavone in caco-2 cells by uhplc-esi-ms/ms and uhplc-esi-q-tof-ms/ms. J. Pharm. Biomed. Anal..

[20] Hroudová J., Singh N., Fišar Z. (2014). Mitochondrial dysfunctions in neurodegenerative diseases: relevance to Alzheimer's disease. BioMed Res. Int..

[21] Kerr J.S., Adriaanse B.A., Greig N.H., Mattson M.P., Cader M.Z., Bohr V.A., Fang E.F. (2017). Mitophagy and Alzheimer's disease: cellular and molecular mechanisms. Trends Neurosci..

[22] Marras C., Beck J.C., Bower J.H., Roberts E., Ritz B., Ross G.W., Abbott R.D., Savica R., Van Den Eeden S.K., Willis A.W., Tanner C.M. (2018). Parkinson's foundation P4 group prevalence of Parkinson's disease across North America. NPJ Parkinsons Dis.

[23] Bose A., Beal M.F. (2016). Mitochondrial dysfunction in Parkinson's disease. J. Neurochem..

[24] Perier C., Bové J., Dehay B., Jackson-Lewis V., Rabinovitch P.S., Przedborski S., Vila M. (2010). Apoptosis-inducing factor deficiency sensitizes dopaminergic neurons to Parkinsonian neurotoxins. Ann. Neurol..

[25] Scarffe L.A., Stevens D.A., Dawson V.L., Dawson T.M. (2014). Parkin and PINK1: much more than mitophagy. Trends Neurosci..

[26] Singh A., Zhi L., Zhang H. (2019 Jan 1). LRRK2 and mitochondria: recent advances and current views. Brain Res..

[27] Elfawy H.A., Das B. (2018). Crosstalk between mitochondrial dysfunction, oxidative stress, and age related neurodegenerative disease: etiologies and therapeutic strategies. Life Sci..

[28] Khan H., Ullah H., Aschner M., Cheang W.S., Akkol E.K. (2020). Neuroprotective effects of quercetin in alzheimer's disease. Biomolecules.

[29] Fiorito V., Chiabrando D., Tolosano E. (2018). Mitochondrial targeting in neurodegeneration: a heme perspective. Pharmaceuticals.

[30] Rahman M.H., Bajgai J., Fadriquela A., Sharma S., Trinh T.T., Akter R., Jeong Y.J., Goh S.H., Kim C.-S., Lee K.-J. (2021). Therapeutic potential of natural products in treating neurodegenerative disorders and their future prospects and challenges. Molecules.

[31] Purohit P.K., Saini N. (2021). Mitochondrial microRNA (MitomiRs) in cancer and complex mitochondrial diseases: current status and future perspectives. Cell. Mol. Life Sci..

[32] Wang (2020). Mol. Neurodegener..

[38] National institue of aging (2019). https://www.nia.nih.gov/health/alzheimers-disease-genetics-fact-sheet.

[41] Yin X., Manczak M., Reddy P.H. (2016). Mitochondria-targeted molecules MitoQ and SS31 reduce mutant huntingtin-induced mitochondrial toxicity and synaptic damage in Huntington's disease. Hum. Mol. Genet..

[42] Sanjari Moghaddam H., Valitabar Z., Ashraf-Ganjouei A. (2018). Cerebrospinal fluid C-reactive protein in Parkinson's disease: associations with motor and non-motor symptoms. Neuromol Med.

[43] Adedara A.O., Babalola A.D., Stephano F., Awogbindin I.O., Olopade J.O., Rocha J.B.T, Whitworth A.J., Abolaji A.O. (2022). An assessment of the rescue action of resveratrol in parkin loss of function-induced oxidative stress in Drosophila melanogaster. Sci Rep..

[44] Waseem Rashid, Shamsi Anas, Kazim N., Syed, Islam A. (2021). An insight Into mitochondrial dysfunction and its implications in neurological diseases. Curr. Drug Targets.

[45] Wang W., Zhao F., Ma X. (2020). Mitochondria dysfunction in the pathogenesis of Alzheimer’s disease: recent advances. Mol. Neurodegen..

[46] Hemachandra Reddy P., Manczak Maria, Kandimalla Ramesh (2017). Mitochondria-targeted small molecule SS31: a potential candidate for the treatment of Alzheimer’s disease. Hum. Mol. Genet..

